# Outbreak of Staphylococcal Food Poisoning from a Military Unit Lunch Party — United States, July 2012

**Published:** 2013-12-20

**Authors:** Nathan S. Teague, Stephanie S. Grigg, Jasmine C. Peterson, Gerardo A. Gómez, Deborah F. Talkington

**Affiliations:** US Army; Div of Foodborne, Waterborne, and Environmental Diseases, National Center for Emerging and Zoonotic Infectious Diseases, CDC

On July 30, 2012, the emergency department at a military hospital was visited by 13 persons seeking care for gastrointestinal illness with onset 2–3 hours after a work lunch party. The hospital responded by opening up temporary evaluation and treatment capacity in primary-care clinics and a progressive-care unit and by diverting one patient to a local civilian hospital. An immediate outbreak investigation was conducted by local military public health personnel with assistance from CDC. Initial epidemiologic analysis implicated “perlo” (a chicken, sausage, and rice dish) and bacterial intoxication as the outbreak mechanism. This enabled public health personnel to 1) recommend no further consumption of perlo and 2) reassure appropriate authorities that no additional ill persons likely would be seeking care and advise that nothing more than supportive care of ill persons likely would be required. After interviewing party attendees, investigators found nine additional persons who met their case definition. Subsequent CDC laboratory analysis of a sample of perlo detected staphylococcal enterotoxin A, supporting the epidemiologic findings. Improper food handling and preparation measures were identified and addressed by the appropriate authorities, who provided additional detailed education on food preparation safety for the persons who prepared the meal.

## Epidemiologic and Environmental Investigation

Immediate steps were taken to interview the 12 patients who received care at the military hospital (one of the 13 who sought care at the emergency department was diverted to a local civilian hospital) using a questionnaire to define the problem, make public health and clinical recommendations as necessary, and assess future patient, hospital, and military installation impact. A case initially was defined as gastrointestinal illness (i.e., nausea, vomiting, abdominal pain, or diarrhea) in a person who had attended the lunch party. Stool specimens were obtained from three persons who met this case definition, and leftover food was collected from all prepared dishes and sent to CDC for analysis. A party attendee list was obtained, and 35 (88%) of 40 attendees were interviewed with the same questionnaire; the other five attendees were not available for interview. The case definition was later modified to include any of the 35 attendees who experienced gastrointestinal illness within 24 hours of the party.

Of the 35 persons interviewed, 22 (62%) met the modified case definition. Among the 22 patients, 19 (86%) had nausea, 15 (69%) vomiting, 17 (77%) diarrhea, 17 (77%) abdominal pain, and 13 (59%) headache. Thirteen (59%) reported feeling chills without fever, four (18%) reported fever with chills. None of the 12 persons who received care at the military hospital had a documented temperature >100.5°F (>38.1°C). Other than the 13 persons who sought care at the military hospital (one was diverted to a civilian hospital), no other person who was interviewed and met the case definition sought medical care. Mean self-reported period to illness onset was 2.1 hours from the time of consumption (Figure), and mean duration of illness was 10.7 hours (range: 1–32 hours).

Illness was associated with eating perlo (risk ratio = 5.7); however, the association did not reach statistical significance (95% confidence interval = 0.9–35.0) (Table). The stool specimens from three patients and samples from the four main dishes (i.e., perlo, chicken wings, pulled pork, and green beans with potatoes) were sent to CDC for laboratory testing for the most likely bacterial organisms based on the epidemiologic investigation (*Staphylococcus aureus, Bacillus cereus,* and *Clostridium perfringens*). Using commercially available rapid detection test kits, laboratory workers tested all of the four main dishes for the presence of staphylococcal enterotoxins (A through E) and *B. cereus* diarrheal toxins (HBL and NHE). The three stool specimens were tested for *C. perfringens* enterotoxin (CPE).

Laboratory testing detected staphylococcal enterotoxin A in the perlo dish, confirming that this outbreak resulted from a staphylococcal intoxication. Evidence of enterotoxin was not found in the other foods. Perlo was cultured for *S. aureus,* and 7.2 x 10^6^ colony forming units/g were isolated. Nucleic acid amplification testing of coagulase-positive isolates detected the enterotoxin gene *sea*, but not *seb, sec, sed, see,* or *seh* (1). Testing for *B. cereus* and *C. perfringens* was negative.

## Food Preparation Findings

Food for the July 30 lunch party was purchased during July 27–28, with the exception of the pork, which was purchased approximately 1 week earlier. All perishable products (chicken thighs, chicken wings, breakfast sausage, and pork) were stored in a freezer.

### Perlo

On July 29, the raw chicken thighs and sausage were defrosted in the microwave. The defrosted chicken thighs were cooked in a stock pot of boiling water. After cooling, the chicken was removed from the thigh bones by hand and placed back into the stock pot. Sausage was cooked in a skillet and added to the pot. Onions and other seasonings were sautéed in the sausage oil and added to the pot. To complete the perlo, rice was added to the stock pot and cooked until all remaining water was absorbed. The pot of cooked perlo then was placed in an unheated oven for approximately 8 hours overnight. On the morning of July 30, the perlo was found to be warm. It was transferred to a slow cooker for reheating for approximately 1 hour on a high setting before transport and consumption.

### Chicken wings

On July 28, the raw chicken wings were defrosted in the refrigerator overnight. On July 29, they were seasoned and placed back in the refrigerator. On the morning of July 30, they were fried and placed in a foil-covered pan for transport and consumption on July 30.

### Pulled pork

The raw pork was defrosted in the microwave on July 29 and cooked overnight in a slow cooker for approximately 8 hours on a low setting before transport and consumption on July 30.

### Green beans with potatoes

On the morning of July 30, the green bean and potato dish was prepared by combining canned green beans and chopped potatoes in a slow cooker on a high setting.

All precooked food was maintained in slow cookers for transport on July 30 and reheated at the venue in separate slow cookers with the exception of the chicken wings, which were placed in a foil container after preparation and never reheated. The food was served buffet style at approximately 11:00 a.m., using utensils owned by the preparer. Throughout the food preparation and food service process, food preparers did not wear gloves. The preparers did not have any open wounds on their hands, and their hands were not tested for any organisms.

### Editorial Note

Staphylococcal food poisoning, one of the most common foodborne illnesses in the United States, is caused by ingestion of one or more preformed staphylococcal enterotoxins. *S. aureus* is able to grow and express enterotoxins in a wide variety of foods (e.g., milk, meat, and egg products; mixed foods; cakes; and ice cream) (2). Intoxication is characterized by rapid onset of nausea, violent and copious vomiting, and abdominal cramping (with or without diarrhea). Fever usually is absent. The incubation period ranges from 30 minutes to 8 hours (3 hours on average), depending on individual susceptibility and the amount of toxin ingested (3). Illness usually is self-limited, resolving in 24–48 hours, and rarely is severe enough to warrant hospitalization (2,4).

What is already known on this topic?

*Staphylococcus aureus* intoxication is a common foodborne illness that usually is not detected or reported because outbreaks are sudden and short-lived, have a low mortality rate, and laboratory confirmation is not obtained. Staphylococcal enterotoxin A is the most common cause of staphylococcal food poisoning.

What is added by this report?

This report describes 22 cases of staphylococcal intoxication associated with a lunch party at a military base. Epidemiologic analysis suggested a preformed enterotoxin in a chicken, sausage, and rice dish. Isolation of *S. aureus* along with identification of staphylococcal enterotoxin A in food confirmed the cause of illness. Rapid detection methods, which are widely available commercially, were used to detect the enterotoxin in food samples, establishing the likely cause of the outbreak before culture results were available.

What are the implications for public health practice?

This report highlights the importance of immediate public health outbreak response, adds to the understanding of food poisoning caused by *S. aureus*, and confirms the need to communicate better food safety practices to both food workers and the general public. Laboratory use of toxin detection kits can provide rapid identification of staphylococcal enterotoxins directly from food and help guide the response of public health authorities.

*S. aureus* produces numerous serologically distinct enterotoxins, (A through V, excluding F) (2). Staphylococcal enterotoxin A is considered the main cause of staphylococcal food poisoning (4). Illness can be caused by ingestion of as little as 20 ng of enterotoxin (2). Although staphylococci commonly are found on environmental surfaces and in various food products, food handlers carrying enterotoxin-producing *S. aureus* in their noses or on their hands are regarded as the main source of contamination (2,5).

Temperature limits for *S. aureus* replication and enterotoxin production are 43°F–118°F (6°C–48°C) and 50°F–115°F (10°C–46°C), respectively. Optimal growth temperatures for *S. aureus* replication and enterotoxin production are 95°F–106°F (35°C–41°C) and 93°F–104°F (34°C–40°C), respectively (2). Staphylococcal enterotoxins are resistant to heat treatment, low pH, and proteolytic enzymes (all of which easily destroy *S. aureus*). Once toxins are produced, they are retained through subsequent food preparation and storage processes and digestive tract ingestion (2). Measures to prevent the proliferation of the *S. aureus* organism therefore are critical.

In this outbreak, the initial source of contamination of the perlo is unknown but might have occurred while the preparer was handling the chicken after it was initially cooked. The overnight storage of the precooked perlo in an unrefrigerated environment was the probable cause of organism proliferation and enterotoxin production. Subsequent rewarming of the perlo for approximately 1 hour the following day did not destroy the heat-stable toxin and might have further increased toxin load.

Toxin detection kits are commercially available and can detect staphylococcal enterotoxins A through E directly from food. The commercial testing platforms usually are passive agglutination or enzyme-linked immunosorbent assays (6). The *B. cereus* and *S. aureus* enterotoxin tests used in this outbreak are only approved for food samples; the test for the *C. perfringens* enterotoxin is approved only for stool specimens. The commercial kits were helpful in the identification of *S. aureus* toxin as the cause of this outbreak and might be useful for state and local public health agencies*.*

In this outbreak, as in many others, poor food handling practices and inadequate refrigeration of foods were identified as the main contributing factors (2,7). Staphylococcal contamination and subsequent intoxication can be prevented by ensuring clean food preparation, storage, and equipment surfaces, and by immediately cooling and storing prepared “potentially hazardous” foods at temperatures below 41°F (5°C). To permit rapid cooling after preparation, food should be stored in small portions in containers that are shallow and loosely covered to facilitate adequate air flow and rapid transfer of heat from the food to the container (8). Consuming food immediately after preparation or removal from refrigeration also is advisable. Finally, handwashing for 20 seconds using soap and water before handling food and food preparation and storage materials is a simple but effective preventive measure to avoid staphylococcal contamination.

## Figures and Tables

**FIGURE:**
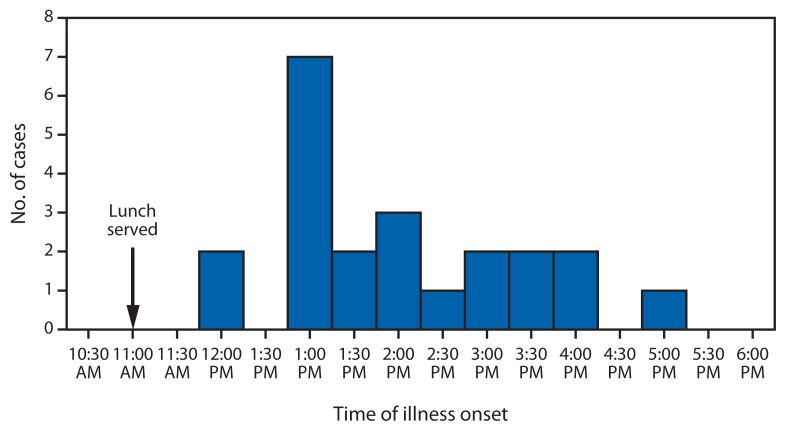
Number of cases (N = 22) of gastrointestinal illness resulting from a military unit lunch party, by time of illness onset — United States, July 2012

**TABLE:** Attack rates and risk ratios comparing cases of gastrointestinal illness (N = 22) associated with a military unit lunch party, by food type — United States, July 2012

Food type	Ate the food			Did not eat the food			Risk ratio	(95% CI)
	
	No. ill	No. well	Attack rate (%)	No. ill	No. well	Attack rate (%)		
Perlo*	21	5	(81)	1	6	(14)	5.7	(0.9–35.0)
Chicken wings	17	11	(61)	5	0	(100)	0.6	(0.5–0.8)
Green beans with potatoes	11	3	(79)	11	8	(58)	1.4	(0.9–2.2)
Pulled pork	17	8	(68)	5	3	(63)	1.1	(0.6–2.0)
Chocolate-covered strawberries	4	4	(50)	18	7	(72)	0.7	(0.3–1.5)
Cake	7	6	(54)	15	5	(75)	0.7	(0.4–1.3)

**Abbreviation:** CI = confidence interval.

* Perlo is a chicken, sausage, and rice dish.
